# Cost-effectiveness analysis of infant pneumococcal vaccination with PHiD-CV in Korea

**DOI:** 10.1080/21645515.2017.1362513

**Published:** 2017-11-08

**Authors:** Xu-Hao Zhang, Oscar Leeuwenkamp, Kyu-Bin Oh, Young Eun Lee, Chul-Min Kim

**Affiliations:** a GSK, Singapore; b Eclipse, Tervuren, Belgium; c GSK, Seoul, Korea; d IMS Health, Seoul, Korea; e Department of Family Medicine, The Catholic University, Seoul St. Mary Hospital, Seoul, Korea

**Keywords:** cost-effectiveness, Korea, PHiD-CV, pneumococcal, vaccine

## Abstract

**Background**: *Streptococcus pneumoniae* and non-typeable *Haemophilus influenzae* (NT*Hi*) can cause invasive pneumococcal diseases (IPD), pneumonia, and acute otitis media (AOM). Both the 10-valent pneumococcal NT*Hi* protein D conjugate vaccine (PHiD-CV) and the 13-valent pneumococcal conjugate vaccine (PCV-13) are included in the National Immunization Program for infants in Korea. This study aimed to evaluate the cost-effectiveness of the 3+1 schedule of PHiD-CV versus that of PCV-13 for National Immunization Program in Korea.

**Methods**: A published Markov model was adapted to evaluate the cost-effectiveness of vaccinating the 2012 birth cohort with PHiD-CV vs. PCV-13 from the Korean government perspective over 10 y. Best available published data were used for epidemiology, vaccine efficacy and disutilities. Data on incidence and direct medical costs were taken from the national insurance claims database. Sensitivity analyses were conducted to explore the robustness of the results.

**Results**: PHiD-CV was projected to prevent an additional 195,262 cases of pneumococcal diseases and NT*Hi*-related diseases vs. PCV-13, with a substantially greater reduction in NT*Hi*-related AOM and a comparable reduction in IPD and community-acquired pneumonia. Parity-priced PHiD-CV generated a health gain of about 844 quality-adjusted life years and a total cost-saving of approximately 4 million United States Dollars (USD) over 10 y. 93% of probabilistic simulations found PHiD-CV 3+1 to be the dominant vaccine option.

**Conclusion**: Compared to PCV-13, PHiD-CV was projected to provide similar prevention against IPD and community-acquired pneumonia but would prevent more cases of AOM. Parity-priced PHiD-CV was anticipated to generate substantial cost-savings and health benefits vs. PCV-13 in Korea.

## Introduction

*Streptococcus pneumoniae* (*S pneumoniae*) is a significant cause of a spectrum of infectious diseases worldwide, and can cause invasive pneumococcal diseases (IPD) such as meningitis and bacteremia, non-invasive lower respiratory tract infections such as pneumonia, and non-invasive upper respiratory tract infections like sinusitis and acute otitis media (AOM).^1^ IPD, pneumonia and AOM may affect all ages; however incidence peaks in the young and the elderly.^2^ *Haemophilus influenzae (H influenzae)*, a gram-negative *coccobacillus* that colonizes the human nasopharynx, is also an important pathogen, particularly in young children.^3^ Non-typeable *Haemophilus influenzae* (NT*Hi)* commonly causes AOM and sinusitis.^4^

*S pneumoniae* is believed to be the most common pathogen of invasive bacterial diseases in children.^5^ There were 2 retrospective multi-center studies investigating the causative agents of invasive bacterial infections in children during 2 different study periods in Korea. The first study was conducted from 1996 to 2005 among subjects aged less than 15 y, involving 18 university hospitals across the country. This study reported that *S pneumoniae* accounted for 23.4% of all invasive bacterial infections. In young children, from 3 months to 5 y of age, 45.3% of the recorded cases were due to the *pneumococcus* pathogen.^6^ Another study was conducted during the period 2006–2010, involving 25 general or university hospitals, and evaluated subjects aged less than 18 y. This study showed that IPD accounted for 23.2% of invasive bacterial infections in Korean children and that 54% of these infections manifested in children aged 3 months to less than 5 y.^7^

According to local experts in the area of pediatric infectious diseases, obtaining an accurate etiological diagnosis of bacterial pneumonia in children is frequently compromised by the practical challenge of collecting an adequate respiratory specimen.^8^ Nevertheless, a retrospective study retrieving records from Korean health insurance databases demonstrated a high burden in terms of hospitalizations and deaths due to pneumonia and showed an increasing trend for all age-groups during the study period of 2002–2005.^9^

A review of the health insurance database in 2004 revealed that the incidence of outpatient AOM was 60.9 per 1,000 population, with the highest rate reported in children aged 1 y where the reported incidence was 736.9 per 1,000 population.^10^ The total cost incurred due to AOM in Korea was estimated to be as high as approximately 559 million United States Dollars (USD)^10^ (all currency converted to USD using monthly average from X-Rates 2015, 1 Korean Won (KRW) = 0.00092 USD^11^). Based on the literature review, the bacterial causes of AOM have remained largely the same for the past century. *S pneumoniae* and *H influenzae* are by far the most common causes of AOM. A review of 23 global AOM etiology studies showed *S pneumoniae* as the dominant strain (59% for less than 1 y old; 43% for 1–4 y old), and *H influenzae* as the second (19% for less than 1 y old; 43% for 1–4 y old).^12^ It is also worthwhile to note that NT*Hi* has become a more important or even dominant pathogen in the era of PCVs, potentially due to replacement reported with 7-valent pneumococcal protein conjugate vaccine (PCV-7).^13-17^

Given the high costs and prevalence of pneumococcal diseases among children, prevention of *S pneumoniae* and NT*Hi* infections is anticipated to improve health status and reduce the burden on the Korean health system. In November 2003, the PCV-7 was introduced as an optional and self-pay vaccination against pneumococcal diseases in Korea.^18^

In March 2010, *Synflorix* (GSK, Belgium), a 10-valent pneumococcal NT*Hi* protein D conjugate vaccine (PHiD-CV), and *Prevnar 13* (Pfizer, USA), a 13-valent pneumococcal conjugate vaccine (PCV-13), were almost simultaneously approved by the Korean Food and Drug Administration for optional vaccination, replacing PCV-7 in the private market. Both vaccines were recommended by the Committee on Infectious Diseases of the Korean Pediatric Society in 2011 and have been included in National Immunization Programs (NIP) for infants in Korea since 2014.^19,20^

Apart from the latest available clinical efficacy and effectiveness data of the 2 Pneumococcal Conjugate Vaccines (PCVs) globally, the health economic aspects of both vaccines may facilitate governmental decision-making regarding vaccination policy and funding allocation for pediatric vaccination programs. Vaccination programs with PCV have been shown to be cost-effective in high-income countries in several previously published studies in Europe and North America. For example, universal infant vaccination with PCV-7, compared with no vaccination and taking herd effects into account, was estimated to be cost-saving in Germany,^21^ and had an estimated cost-effectiveness ratio of 7,500 USD per life-year saved in the United States (US),^22^ £4,360 per life-year gained in the United Kingdom (UK),^23^ and €5,500 per quality-adjusted life-year (QALY) gained in Sweden.^24^ More recently, PHiD-CV and PCV-13 were both estimated to have more favorable cost-effectiveness ratios than PCV-7 in Australia, although the results were sensitive to changes in assumptions about herd protection, serotype protection, otitis media efficacy and vaccination cost.^25^ In Japan, vaccination with PHiD-CV was also estimated to be cost-saving compared with PCV-13, from the healthcare provider and societal perspectives.^26^ A review of 15 cost-effectiveness studies on PCV published between 2002 and 2006 found that PCV vaccination could generally be considered attractive in developed countries, although the results varied considerably between studies.^27^ Factors contributing to the variation in results included differences in assumptions about vaccine efficacy parameters and disease incidence.^27^ A comparative analysis of different models used in PCV cost-effectiveness studies found that vaccine efficacy, vaccine cost, vaccine coverage, serotype coverage and disease burden were influential parameters.^28^ There is currently a lack of published data in Korea, and therefore Korea-specific data will be valuable to inform decisions locally.

Therefore, it was considered relevant to evaluate the cost-effectiveness of the available PCVs in the Korean context.^29^ Although the conclusions of such an analysis are not directly transferable to other jurisdictions due to variations in disease epidemiology, clinical practice including treatment patterns, healthcare systems, vaccination programs, healthcare utilization and medication costs, the inferences of performed cost-effectiveness evaluation may bear a broader significance.

The purpose of this study is to evaluate the epidemiological and economic consequences of including the PCV(s) recently added to the NIP, supplementing the current standard of care for managing cases of pneumococcal diseases in Korea. Specifically, this paper will focus on the comparison of costs and health benefits and present the results of a cost-effectiveness analysis for Korea. In addition, this study was aimed to compare 3+1 NIPs involving 3+1 schedules of PHiD-CV and PCV-13 vaccination modalities.

## Results

Table 1 presents the estimated impact over 10 y on the disease burden of a 3+1 vaccination program involving PHiD-CV vs. that of PCV-13 for the 2012 birth cohort in Korea. It was projected that PHiD-CV 3+1 would prevent a comparable number of IPD and pneumonia cases, but PHiD-CV would establish a substantially greater reduction in the number of AOM cases (n = 195,279), compared with PCV-13. Furthermore, the number of simulated deaths was identical for the compared vaccines.

**Table 1.** Impact of 3+1 vaccination strategies on disease burden in Korea over 10 y.

Number of cases	PCV-13 3+1 (A)	PHiD-CV 3+1 (B)	PHiD-CV 3+1 vs. PCV-13 (3+1) (B-A)
IPD hospitalization (meningitis + bacteremia)	189	207	18
Community-acquired pneumonia hospitalization/GP consultation	1,343,600	1,343,600	0
AOM outpatient and procedures including myringotomy and tube placement (acute episode)	2,114,009	1,918,730	−195,279
Death	10,643	10,643	0

AOM: acute otitis media, GP: general practitioner, IPD: invasive pneumococcal diseases, PCV-13: 13-valent pneumococcal conjugate vaccine, PHiD-CV: 10-valent pneumococcal Non-typeable *Haemophilus influenzae* protein D conjugate vaccine.

The estimated financial projections for the PHiD-CV 3+1 vaccination program compared with the PCV-13 3+1 strategy at price parity of 51.2 USD/dose are presented in Table 2. Results showed that the total discounted savings with the PHiD-CV 3+1 vaccination program compared with the PCV-13 3+1 program were projected at approximately 4 million USD.

**Table 2.** Economic impact of compared vaccination strategies until the set analytical time horizon of 10 y.

Cost component (in million USD)	PCV-13 3+1 (A)	PHiD-CV 3+1 (B)	PHiD-CV 3+1 vs. PCV-13 3+1 (B-A)
Vaccination	98.25	98.25	0
Acute episodes			
IPD (meningitis + bacteremia)	1.28	1.40	0.12
Community-acquired pneumonia	388.72	388.72	0
AOM (outpatient and procedures including myringotomy and tube placement)	47.26	42.50	−4.76
Total costs (undiscounted)	535.51	530.87	−4.64
Total (discounted)^*^	456.94	452.92	−4.02

* Discount rate: 5%^37^.

AOM: acute otitis media, IPD: invasive pneumococcal diseases, PCV-13: 13-valent pneumococcal conjugate vaccine, PHiD-CV: 10-valent pneumococcal Non-typeable *Haemophilus influenzae* protein D conjugate vaccine, USD: United States Dollars.

The vaccination costs of parity-priced vaccines were 98.3 million USD. Over a period of 10 y, PHiD-CV would generate total savings of about 4.7 million USD in terms of direct costs in conjunction with AOM, compared with PCV-13. On the other hand, PHiD-CV would generate 122,360 USD additional direct costs in association with IPD. Overall, these results translated into discounted cost savings of about 4 million USD for PHiD-CV compared with PCV-13, as shown in Table 2.

### Cost-effectiveness analysis

At price parity, PHiD-CV 3+1 compared with PCV-13 3+1 is predicted to generate overall cost savings of about 4 million USD and an incremental health benefit of approximately 844 QALYs cumulated over a 10-y time period, indicating that PHiD-CV constitutes a dominant vaccine option (Table 3).

**Table 3.** Cost-effectiveness analysis until the set analytical time horizon of 10 y.

Costs (in million USD) and health benefit (QALYs)	PCV-13 3+1	PHiD-CV 3+1	PHiD-CV 3+1 vs. PCV-13 3+1
Total costs (discounted)	456.94	452.92	−4.02
Total QALYs (discounted)	3,439,060	3,439,904	844
ICER (discounted)	*Dominant*		

ICER: incremental cost-effectiveness ratio, PCV-13: 13-valent pneumococcal conjugate vaccine, PHiD-CV: 10-valent pneumococcal Non-typeable *Haemophilus influenzae* protein D conjugate vaccine, QALYs: quality-adjusted life years, USD: United States Dollars.

### Sensitivity analyses

**Figure 1.**
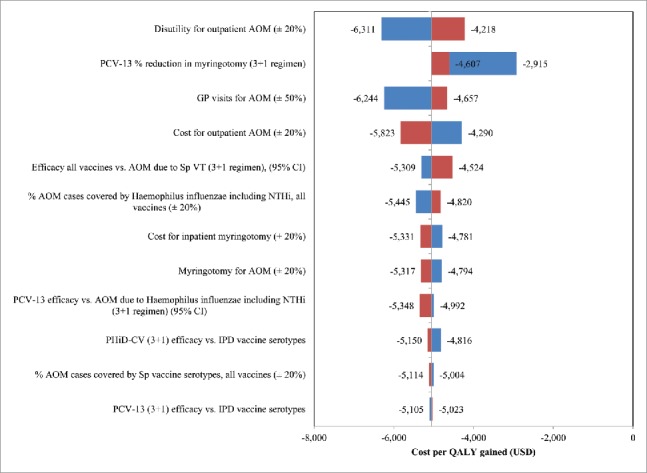
The top 10 parameters that are most influential in accordance with the performed one-way sensitivity analyses. AOM: acute otitis media, CI: confidence interval, GP: general practitioner, IPD: invasive pneumococcal diseases, NTHi: Non-typeable Haemophilus influenzae, PCV-13: 13-valent pneumococcal conjugate vaccine, PHiD-CV: 10-valent pneumococcal Non-typeable Haemophilus influenzae protein D conjugate vaccine, Sp: Streptococcus pneumoniae, USD: United States Dollars, VT: Vaccine Type, QALY: quality-adjusted life year.

Based on the extensive one-way sensitivity analyses, it was shown that our model predictions were robust. Although the variations explored in the parameters did not have a significant impact on the conclusion, the results were sensitive to parameters related specifically to AOM (Fig. 1).

The probabilistic sensitivity analyses showed that PHiD-CV 3+1 was cost-saving in 93% and cost-effective in 93.4% of the simulations, compared with PCV-13 3+1.

## Discussion

The goal of this economic evaluation was to assess the cost-effectiveness of a PHiD-CV 3+1 vaccination program vs. a PCV-13 3+1 vaccination strategy, from the perspective of the Korean government. Price parity at 51.2 USD per dose (equating to the average of the local price points of PHiD-CV and PCV-13) was assumed.

Based on the evaluations, both vaccines showed comparable reductions in the number of IPD cases (pneumococcal meningitis and pneumococcal bacteremia) and community-acquired pneumonia. Vaccination with PHiD-CV was found to prevent an additional 195,279 cases of AOM, including tube placement, over the 10-y analytical time horizon adopted, implying that PHiD-CV can potentially reduce the number of antibiotic prescriptions to children in Korea. A reduction in the number of those prescriptions could be instrumental in managing the evolution of antibiotic resistance among Korean children. The incremental benefit of PHiD-CV in preventing AOM, including myringotomy and tube placement, would translate into cost savings of about 4 million USD and an additional health gain of about 844 QALYs, cumulated over 10 y.

One-way sensitivity analyses consistently revealed that the model results were more sensitive to AOM related parameters. This could be explained by the high incidence of AOM and tube placement procedures in Korea compared with IPD and, to a lesser extent, pneumonia. According to Palmu *et al.* (2014)^30^ high incidence of AOM can be more important than IPD and pneumonia from an economic point of view. This would apply particularly in conditions where the incidence of IPD is profoundly reduced by virtue of deployed pneumococcal vaccination programs in infants.

Our findings are consistent with those of other published cost-effectiveness analyses comparing identical vaccination programs of PHiD-CV and PCV-13 in developed countries. PHiD-CV was found to be cost-saving compared with PCV-13 from the societal and/or provider perspectives regardless of variations in terms of epidemiological conditions and healthcare systems. A recent publication by Shiragami *et al.* (2014)^26^ addressed the comparison of PHiD-CV 3+1 and PCV-13 3+1 in the context of a pediatric NIP in Japan over an analytical time horizon of 5 y. The model projected that vaccination with PHiD-CV would result in total cost savings of 16 million or 33 million USD from the provider and societal perspectives, with additional 433 QALYs gained compared with PCV-13.^26^

By *et al.* (2012)^31^ used a Markov cohort model to compare PHiD-CV 2+1 vs. PCV-13 2+1 strategy in Sweden, taking a societal perspective. It was found that the PHiD-CV strategy would generate additional 45.3 QALYs with substantial cost-savings estimated at close to 9.3 million USD for a cohort of 112,120 children.^31^ Robberstad *et al.* (2011)^32^ also used a Markov model to evaluate the cost-effectiveness of pneumococcal conjugate vaccines (PCV-7, PCV-13 and PHiD-CV) for a birth cohort (n = 61,152) in Norway. PHiD-CV was associated with cost-savings of close to 4.15 million USD with an additional 49 QALYs gained.^32^

The modeling published by Knerer *et al.* (2012),^33^ which forms the basis of the current cost-effectiveness analysis, found that PHiD-CV is also a dominant strategy when compared with PCV-13, offering additional savings of 9.2 million USD for a birth cohort of approximately 348,000 newborns in Canada, and additional savings of close to 7.2 million USD in the UK for a birth cohort of approximately 772,500 newborns.^33^

It is noteworthy that a conservative approach was taken in the reported analyses comparing PHiD-CV with PCV-13. Optimal assumptions were applied for PCV-13, due to the lack of efficacy information from randomized controlled trials about PCV-13 in IPD, pneumonia and AOM. Based on Clinical Otitis Media & Pneumonia Study (COMPAS), we assumed that PHiD-CV demonstrated reported efficacy against NT*Hi*-related AOM and AOM-associated tube placement. Long-term sequelae and reductions in antibiotic prescriptions were not included in the analysis as locally relevant data were lacking.^30^ In view of the above, the cost savings and incremental QALYs predicted to be generated by PHiD-CV in comparison with PCV-13 are likely to be underestimated, resulting in a conservative cost-effectiveness analysis.

As is the case for all published modeling exercises, the current analysis has several limitations. In the first place, the herd effect in conjunction with IPD was not addressed in the current model due to the lack of knowledge or published data with regards to the potential differences in herd protection induced by each vaccine. It was assumed that the compared vaccines would induce the same herd protection effect, based on similar efficacy profiles, and thus herd protection would not have an impact on the final model results. The same argument applies to serotype replacement, which exerts an effect opposite to that of herd protection. Secondly, we used the best data locally available wherever possible in the current analysis. However, some specific clinical epidemiology variables were hard to estimate due to the lack of an active surveillance system in Korea. Clinical experts (infectious disease specialists and ear, nose and throat specialists) were consulted to provide the best estimates possible for the number of pediatric IPD cases and to validate the incidence of AOM involving tube placement for pediatric patients in Korea. Likewise, disutility weights were not available for the local population, so we used published data in the analysis and have explored the uncertainty and impact in the sensitivity analyses. The current model is an adaptation of a previously published model.^33^ The main adaptation we made in the current analysis was to exclude the sequelae component from the original model, due to the lack of reliable data on sequelae related to IPD, pneumonia and AOM from the local insurance database. However, we would not expect the exclusion of sequelae to change our results significantly, as the incidence of sequelae are in general very low and has only demonstrated marginal effects on the incremental cost-effectiveness ratios (ICER) in previously published studies.^26,32,33^

Extensive sensitivity analyses were conducted to explore the extent to which the model outcomes are influenced by uncertainty in the information used to populate the model, and to obtain insight into the robustness of the results and the conclusions based thereon. Based on the sensitivity analyses performed, it can be put forward that the model results are robust and it can be inferred that PHiD-CV represents a cost-saving vaccination modality also offering a health benefit vs. PCV-13.

Parity-priced PHiD-CV 3+1 vs. PCV-13 3+1 was predicted to offer comparable prevention in terms of IPD and pneumonia and a substantial reduction in the number of AOM cases, generating cost savings of about 4 million USD and a health benefit gain of approximately 844 QALYs over a time period of 10 y. As a result, parity-priced PHiD-CV would be dominant over PCV-13 taking the perspective of the Korean government.

## Materials and methods

### Model structure

A published Markov cohort model formed the basis, and was adapted to simulate the epidemiological burden of pneumococcal and NT*Hi*-related diseases in Korea^33^ and to perform cost-effectiveness analyses.

**Figure 2.**
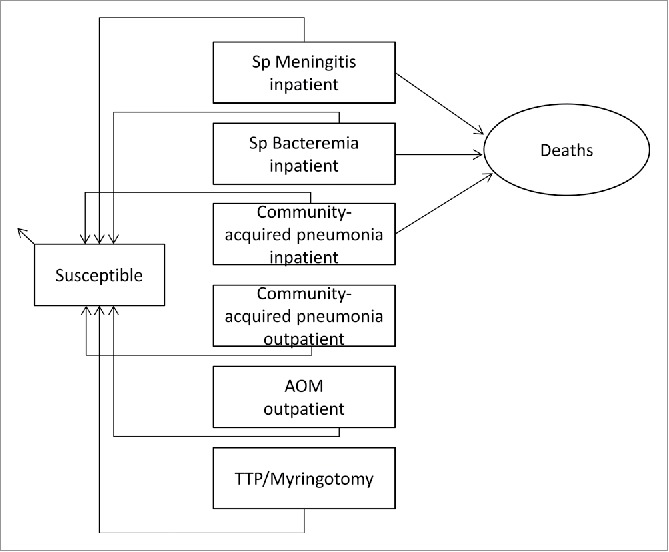
Markov Cohort Model flow diagram. Rectangles represent mutually exclusive health states. Age-specific incidences are applied monthly to the susceptible population. Circles (sequelae and death) and small arrow (natural death) is the proportion of the population removed from the model. Costs and benefits are computed monthly and aggregated over the time horizon indicated for the analysis. AOM: Acute Otitis Media, Sp: Streptococcus pneumoniae, TTP: Tympanostomy Tube Placement.

The flowchart for the Markov cohort model is shown in Fig. 2. The model accommodates IPD including meningitis and bacteremia; community-acquired pneumonia and AOM represent the mutually exclusive disease states. In the Markov cohort model, the individuals of the birth cohort can move to one of the disease conditions or die at a particular age, in accordance with the applicable transition probabilities in conjunction with respective disease states and the probability of dying due to acquired disease conditions or another cause. Based on the applicable efficacy during each monthly cycle of the model, the probability of moving to a specific health state or dying was estimated by taking into account the disease-specific incidence rates for IPD, community-acquired pneumonia and AOM, case fatality ratios (when applicable), and the probability of natural death. Costs and health benefits were accumulated across the model cycles until the set analytical time horizon of 10 y.

The model simulated the health outcomes and costs of vaccination for the 2012 birth cohort comprising 484,550 Korean newborns.^33,34^ In the base-case analysis, the time horizon was limited to 10 y for multiple reasons. Uncertainty regarding the serotype distribution is expected to increase over time. After the introduction of the PCV, vaccine efficacy (VE) was assumed to last for 10 y^35^ and pneumococcal disease caused the highest burden in infants and young children.^5^ This model assumed the ramp-up of VE, achieving full efficacy after the final vaccination at the age of 12–15 months. VE will then begin to decline at the age of 3 y and will be lost at the age of 10 y, as illustrated by De Wals *et al.* 2009.^36^ In the current model, it was assumed that the 2 vaccines would provide similar herd protection effects.^31^ On the basis of this consideration, herd protection was not addressed in the model as it would not affect the results of comparing the 2 vaccines.

Costs and QALYs specific to each health state were estimated and accumulated for 10 y, representing the set analytical time horizon. Costs were presented in USD for the benefit of international readers and all costs were standardized to year 2012. Health effects and costs were both discounted at 5% per annum in the base-case analysis.^37^

ICER were computed, taking into account the differential costs and benefits of the 3+1 schedules of both PHiD-CV and PCV-13. A strategy was considered dominant if it cost less and gained more QALYs. PHiD-CV was concluded to be cost-effective if the ICER was below the Korean Gross Domestic Product per capita, and not cost-effective if the ICER was greater than the Gross Domestic Product per capita of Korea in 2012 (approximately 25,343 USD).^34^

All analyses were conducted from the perspective of the Korean government. Consequently, only direct medical costs (e.g. hospitalization, inpatient/outpatient diagnostic tests and procedures, medication/vaccine costs, logistics costs, and healthcare professionals' fees) were included.

### Epidemiology parameters

A publicly available de-identified random sample of 1.3 million patients from the 2011 Health Insurance Review Agency (HIRA) database was accessed to retrieve incidence rates and costs based on appropriate International Statistical Classification of Diseases and Related Health Problems (ICD)-10 codes identifying anonymized patients of interest who experienced the disease conditions.^38^ Applied codes were consolidated by local experts.^38^ Consecutive records of the same patient within a 30-day interval were considered as belonging to the same disease episode both for inpatient and outpatient visits.

According to local clinical experts, the IPD incidence rates of 0.29 per 100,000 for pneumococcal meningitis and 0.44 per 100,000 for pneumococcal bacteremia derived from the random sample concern underestimates potentially due to the very low incidence rates and the small size of the random sample accommodating 3% of the entire population.^38^ Incidence rates derived on basis of cases coded as meningitis or bacteremia in the database are likely underestimates as not all cases are coded in terms of these disease conditions due to a deviating primary diagnosis. Based on expert consultation, the incidence rate of pneumococcal meningitis for patients under 10 y was obtained assuming that 27% of the total number of unspecified bacterial meningitis and pneumococcal meningitis cases occurred in this age range in 2011 as reported by the HIRA.^38,39^ In Korean practice, cases of pediatric pneumococcal bacteremia are commonly managed and documented in first instance in the emergency room;^38,40^ therefore, the incidence rate of pediatric pneumococcal bacteria in children under 10 y was based on the proportion of all emergency room visits reported for this age range at the HIRA website. Natural death rates by age group were retrieved from the national life tables of the Korean Statistical Information Service.^34^ The pneumonia and AOM information from the database was validated by specialists who ascertained that the random sample from HIRA yields reliable estimates for the local incidence rates which are high relative to those of IPD. Further, the criteria applied for diagnosing AOM vary among physicians and the threshold for getting access to the Korean healthcare system is low. The latter may account for the high incidence rate of AOM in Korea compared with other countries in the region, e.g., Taiwan.^41^

For children/infants, only tube placement procedures were considered in conjunction with AOM. The epidemiological data derived for IPD, community-acquired pneumonia and AOM are reported in Table 4.

**Table 4.** Epidemiological data for individuals up to 10 y of age or otherwise for the age groups specified.

Category	Value Estimates	References
Pneumococcal meningitis		
Hospitalization rate per 100,000 population (age group)	1.86 (< 10 years)	^38,39^
Case fatality rate, % (age group)	9.50 (< 10 years)	^8^
Pneumococcal bacteremia		
Hospitalization rate per 100,000 population (age group)	2.64 (< 10 years)	^38,40^
Case fatality rate, % (age group)	5.60 (< 10 years)	^6^
Community-acquired pneumonia		
GP consultation rate per 100,000 population (age group)	12,458 (< 1 year); 32,543 (1 year); 49,942 (2 years); 42,616 (3 years); 36,607 (4 years); 15,288 (5–9 years)	^38^
Hospitalization rate per 100,000 population (age group)	400 (< 1 year); 12,378 (1 year); 11,222 (2 years); 7,893 (3 years); 5,963 (4 years); 2,566 (5–9 years)	^38^
Case fatality rate, % (age group)	4.90 (< 10 years)	^6^
AOM		
GP consultation rate per 100,000 population (age group)	41,106 (< 1 year); 74,464 (1 year); 94,349 (2 years); 79,164 (3 years); 66,521 (4 years); 26,642 (5–9 years)	^38^
Tube placement in hospital setting per 100,000 population (age group)	333.48 (< 10 years)	^38^

AOM: acute otitis media, GP: general practitioner.

### Vaccine efficacy

A ramp-up of efficacy level by dose was assumed in the model with establishment of full efficacy at the last dose administered at 12–15 months of age.^42^ VE against IPD was calculated from local serotype distribution (based on the latest 2011–2013 multi-center IPD surveillance in Korean children) and serotype-specific efficacy of each vaccine.^43^ VE was assumed to begin to decline at the age of 3 y, with efficacy completely lost by the age of 10 y.^36^ The VE assumptions used in the model are summarized by serotype in Table 5.

**Table 5.** Efficacy assumptions for IPD serotypes and vaccine efficacy against all-cause pneumonia and AOM for 2 vaccines.

Category	Value	References
IPD (by serotype)		
1, 4, 5, 7F, 18C (serotype prevalence: 0.0% )	0.947 (PHiD-CV); 0.947 (PCV-13)	^45^^*^
3 (serotype prevalence: 0.0%)	0.000 (PHiD-CV); 0.260 (PCV-13)	^51^
6A (serotype prevalence: 5.3%)	0.760 (PHiD-CV); 0.947 (PCV-13)	^45^^*^
6B, 9V, 14 (serotype prevalence: 1.3%)	0.947 (PHiD-CV); 0.947 (PCV-13)	^45^^*^
19A (serotype prevalence: 32%)	0.720 (PHiD-CV); 0.947 (PCV-13)	^48,50^
19F, 23F (serotype prevalence: 2.7%)	0.947 (PHiD-CV); 0.947 (PCV-13)	^45^^*^
Other (serotype distribution: 53.3%)	0.000 (PHiD-CV); 0.000 (PCV-13)	Assumption
Community-acquired pneumonia		
Outpatient setting	0.073 (PHiD-CV); 0.073 (PCV-13)	^53^
Inpatient setting	0.234 (PHiD-CV); 0.234 (PCV-13)	^53^
AOM (by cause)		
Vaccine serotypes (10 most common pneumococcal serotypes)	0.699 (PHiD-CV); 0.699 (PCV-13)	^53^
Serotype 3	0.000 (PHiD-CV); 0.000 (PCV-13)	Assumption
Serotype 6A	0.637 (PHiD-CV); 0.699 (PCV-13)	^53,60^
Serotype 19A	0.531 (PHiD-CV); 0.699 (PCV-13)	^45,53^
Non-vaccine serotypes	−0.330 (PHiD-CV); −0.330 (PCV-13)	^14^
NT*Hi*-related	0.215 (PHiD-CV); −0.110 (PCV-13)	^14,15,53^

* The data are extrapolated from reference.^45^

AOM: acute otitis media, IPD: invasive pneumococcal diseases, PCV-13: 13-valent pneumococcal conjugate vaccine, PHiD-CV: 10-valent pneumococcal Non-typeable *Haemophilus influenzae* (NT*Hi*) protein D conjugate vaccine.

On basis of the 3+1 diphtheria toxoid, tetanus toxoid, and acellular pertussis (DTaP) coverage rate of 99% established in 2013 in Korea, it was assumed in the base-case scenario that 99% of the modeled birth cohort would be vaccinated and that all vaccinated children would comply with the scheduled 3+1 doses.^44^ Since the vaccine coverage applies equally to the vaccines compared, the assumed vaccine coverage would have no effect on the estimation of the ICER.

VE against IPD was calculated as a sum product of local serotype distribution (based on the latest 2011–2013 multi-center IPD surveillance information for Korean children) and serotype-specific effectiveness reflected in Table 5 for the comparison of 2 vaccines.^43^ Serotype-specific effectiveness data were largely extrapolated from estimates of VE obtained from a Center for Disease Control and Prevention (CDC) case-control study conducted in the United States for PCV-7 and reported by Whitney *et al.* (2006).^45^ It was assumed that the 10 common types covered by both vaccines (i.e.: 1, 4, 5, 6B, 7F, 9V, 14, 18C, 19F, 23F) would confer 94.7% protection, which represents the weighted average of the serotype-specific estimates for at least 1 vaccine dose and the 7 serotypes included in PCV-7.^45^ Cross-protection for 6A of PHiD-CV was estimated at 76%, based on the same study.^45^ PHiD-CV also elicits cross-reactive functional antibodies against serotype 19A.^46^ In July 2015, *Synflorix* (GSK, Belgium) received a positive opinion from the European Medicines Agency (EMA) to include the 19A immunological and effectiveness data in the label of this vaccine.^47^ Post-marketing surveillance studies in Quebec and Finland^30,48^ have revealed marked impact of PHiD-CV on 19A, with the latest effectiveness study in Brazil demonstrating an effectiveness estimate of 82.2% for 19A.^49^ Conservatively, a vaccine effectiveness of 72% was assumed for PHiD-CV in the base-case scenario as per the latest published Quebec data^48^ and a hypothetical upper limit of 94.7% (average of PCV-7 VE data, which is even higher than the highest reported VE efficacy of PCV-13 3+1 schedule from the United States [90%])^50^ for PCV-13. In this context, it should be noted that the Quebec study evaluating the compared vaccines constitutes the only source providing effectiveness data for these 2 vaccines. The Quebec study assessing the effectiveness of pneumococcal conjugate vaccines revealed similar VE for PHiD-CV (VE = 72% [95% Confidence Interval: 24, 89]), and PCV-13 (VE = 74% [95% Confidence Interval: 11, 93]).^48^ We have assumed 82.2% (19A effectiveness data of PHiD-CV) in the sensitivity analyses.^49^ It is important to note that vaccine effectiveness against serotype 3 has shown conflicting and hyporesponsive results on the impact of IPD in studies from different countries.^51,52^ As serotype 3 is not circulating in the pediatric population in Korea based on current surveillance data,^43^ effectiveness against serotype 3 would not have any impact on the final cost-effectiveness result.

For PHiD-CV, the COMPAS study reports an efficacy of 23.4% against inpatient pneumonia or consolidated community-acquired pneumonia requiring hospitalization and an efficacy of 7.3% against outpatient pneumonia or suspected community -acquired pneumonia commonly managed in an outpatient setting.^53^ These estimates were also applied to PCV-13 in the model due to the lack of reported estimates for this vaccine.^53^

The overall effectiveness against AOM of PHiD-CV has been demonstrated in the latest double-blinded randomized control trial, COMPAS.^53^ So far, there is not sufficient data available on the overall AOM efficacy or effectiveness or pathogen-based AOM efficacy of PCV-13. There is also no evidence to guide projections of the overall AOM efficacy for PCV-13 based on PCV-7 data (Finnish Otitis Media [FinOM]).^14^ Therefore, the vaccine efficacy against AOM was estimated based on efficacy against pneumococcal vaccine and non-vaccine serotype disease and efficacy against disease caused by NT*Hi*. The serotype distribution of *S. pneumoniae* strains was based on a small local study conducted from 2001 to 2006 (N = 54) which cultured the ear discharge in children with otitis media,^54^ as there were very few etiology data on AOM in Korea and results were not available to us.

Due to the lack of recent publications showing the vaccination impact of 2^nd^ generation PCVs on AOM locally, local experts suggested using data from the US, which was based on a 7-y prospective study from June 2006 to August 2013 (PCV-13 was introduced in the US in 2010) of 619 children, in which middle ear fluid cultures obtained by tympanocentesis were collected on children with AOM.^55^ Based on the US data, the model assumed that 28.7% of AOM cases were attributable to *S. pneumoniae* and that 37% of AOM cases were attributable to NT*Hi*.^55^ This US study also reported a close correlation of nasopharyngeal cultures with middle ear fluid cultures. In Korea, there was a clinical epidemiology study of the characteristics of NT*Hi* isolated from the nasopharynx of young children with AOM conducted from January 2011 to March 2012 in 7 hospitals in Korea.^56^ The study reported that for 419 subjects, 136 *H. influenzae* cases (32.5%) were isolated and all of them were NT*Hi*.^56^ The NT*Hi* proportion reported in Korea (32.5%) was very similar to the number (37%) reported in the US study.

For PHiD-CV, data from COMPAS were used for VE against AOM caused by *S pneumoniae* (VE= 69.9%) and NT*Hi*-related AOM (VE= 21.5%).^53^ For PCV-13, pathogen- and serotype-specific VE estimates from the randomized controlled FinOM vaccine trial involving PCV-7 (VE= 57.2% vaccine type [VT] AOM; VE= −11% NT*Hi* AOM) were taken into account in the model.^14^

Maximal effectiveness was calculated for each vaccine using the following equation:^33^

VE_max_ =: VE_VT_ × % of AOM cases due to vaccine serotypes + VE_NVT_ × % of AOM cases due to non-vaccine serotypes + VE_NTHi_ × % of AOM cases due to NT*Hi*
*VE_max_* =: *maximal efficacy*
*VE_VT_* =: *vaccine efficacy against vaccine serotypes*
*VE_NVT_* =: *vaccine efficacy against non-vaccine serotypes, and*
*VE_NTHi_* =: *vaccine efficacy against disease caused by NTHi*

### Disutility weights

Due to the lack of pneumococcal disease-related disutility weights specifically for the Korean population, disutility weights published for other geographies were assumed in the current analyses.^33,57-59^ Table 6 displays the disutility applied in the model for IPD, pneumonia and AOM. Duration of events included only acute episodes. Long-term sequelae were not included due to a lack of local data.

**Table 6.** Disutility weights.

Disease condition	Disutility	References
Meningitis (inpatient)	0.023	^57^
Bacteremia (inpatient)	0.008	^57^
Pneumonia (inpatient)	0.008	^57^^(Assumption)^
Pneumonia (outpatient)	0.006	^57^
AOM (outpatient)	0.005	^59^
AOM (procedures including myringotomy or tube placement)	0.005	^59^^ (Assumption)^

AOM: acute otitis media.

### Costs

Although there is a difference of approximately 16% between the reimbursed price points of PCV-13 (55 USD per dose) and PHiD-CV (47 USD per dose),^43^ price parity was assumed to evaluate cost-effectiveness based on medical evidence alone. In the base-case scenario, 51.2 USD represents the average of current price points for both vaccine formulations. In addition, a logistics fee of 8% and an administration fee of 16.6 USD per dose were taken into account.

Direct medical cost data for the acute episodes, presented in Table 7, were based on the local HIRA data from 2011 and updated to 2012 using the consumer price index of health items.^34,38^ Due to a lack of suitable cost estimation of long-term IPD sequelae in the current analysis, long-term IPD sequelae were excluded from the analysis. This was expected to have a negligible impact on the outcome of the evaluations performed, due to the similarity of the compared vaccines in terms of effectiveness against IPD and the low prevalence of such complications.

**Table 7.** Costs per disease condition.

Disease condition	Value (USD)	References
Pneumococcal meningitis –inpatient	11647.32	^38^
Pneumococcal bacteremia – inpatient	5432.35	^38^
Pneumococcal bacteremia – outpatient	107.13	^38^
Community-acquired pneumonia – inpatient	1716.43	^38^
Community-acquired pneumonia – outpatient	37.40	^38^
Tube placement (pediatric group, including general anesthesia, aged ≤ 10 y old)	506	^38^
AOM GP consultations	19.27	^38^

AOM: acute otitis media, GP: general practitioner, USD: United States Dollars

### Sensitivity analyses

Extensive one-way sensitivity analyses were performed to explore the robustness of the model results and conclusions to changes in parameters. One of 3 methods was used, depending on the parameter: estimates were varied for all age groups simultaneously by ± 20% (or ± 50% where under-reporting of incidence was suspected) of the base-case value; data were varied in accordance with the reported 95% confidence intervals; or a weighted average of studies was calculated to derive suitable values. A probabilistic sensitivity analysis (PSA) was also performed by recording the results of 1,000 Monte Carlo simulations, each of which simultaneously sampled the model's input parameters from the applied appropriate probability distributions (Dirichlet distribution for IPD serotype distribution, triangular distribution for disease incidence and costs; lognormal distribution for vaccine efficacy; and β distribution for disutility values).
